# Diversity and regulatory impact of copy number variation in the primate *Macaca fascicularis*

**DOI:** 10.1186/s12864-017-3531-y

**Published:** 2017-02-10

**Authors:** Andreas R. Gschwind, Anjali Singh, Ulrich Certa, Alexandre Reymond, Tobias Heckel

**Affiliations:** 1 Center for Integrative Genomics, University of Lausanne, Lausanne, Switzerland; 2 Swiss Institute of Bioinformatics SIB, Lausanne, Switzerland; 3 Roche Pharmaceutical Research and Early Development, Roche Innovation Center Basel, 4070 Basel, Switzerland

**Keywords:** Cynomolgus monkey, CNV, Gene expression, eQTL, Olfactory receptors

## Abstract

**Background:**

Copy number variations (CNVs) are a significant source of genetic diversity and commonly found in mammalian genomes. We have generated a genome-wide CNV map for Cynomolgus monkeys (*Macaca fascicularis*). This crab-eating macaque is the closest animal model to humans that is used in biomedical research.

**Results:**

We show that Cynomolgus monkey CNVs are in general much smaller in size than gene loci and are specific to the population of origin. Genome-wide expression data from five vitally important organs demonstrates that CNVs in close proximity to transcription start sites associate strongly with expression changes. Among these eQTL genes we find an overrepresentation of genes involved in metabolism, receptor activity, and transcription.

**Conclusion:**

These results provide evidence that CNVs shape tissue transcriptomes in monkey populations, potentially offering an adaptive advantage. We suggest that this genetic diversity should be taken into account when using Cynomolgus macaques as models.

**Electronic supplementary material:**

The online version of this article (doi:10.1186/s12864-017-3531-y) contains supplementary material, which is available to authorized users.

## Background

Copy number variations (CNVs) are genetic differences in the normal population displayed as microscopically invisible deletions or amplifications of stretches of genomic DNA ranging from 1 kilobase up to the megabase scale [1]. CNVs are commonly found in the genomes of humans [2], primates [3], rodents [4], or arthropods like *Drosophila melanogaster* [5]*.* In humans, more than 6.2 million different CNVs mapping to ~500’000 genomic regions have so far been identified [6]. They significantly contribute to genetic variation, covering more nucleotide content per genome than single nucleotide polymorphisms (e.g. approximately 0.8% of the length of the human genome differs between two human individuals) [7]. Furthermore CNVs exhibit a higher per-locus mutation rate than single nucleotide polymorphisms (SNPs) [8]. Since CNVs can reside in genomic regions harboring genes they can alter gene dosage, disrupt coding sequences or modify the level and timing of gene expression for genes within the CNV [9, 10] and on its flanks [4, 11–15]. These effects of CNVs are difficult to understand and not necessarily predictable, but relevant for many diseases [16–19] and pharmacological responses like in the case of CYP2D6 CNVs [20].

Cynomolgus monkeys (*Macaca fascicularis*) are well-established translational models for biomedical research and drug testing. These non-human primates are one of the closest animal model to humans with high genetic similarity (~93% in nucleotide sequence identity), similar anatomies, and very similar physiologies [21–23]. These animals offer great promise as models for many aspects of human health and disease. Cynomolgus monkeys are outbred species, caught in the wild in many different places of peninsular Southeast Asia, the Philippines, and Mauritius, and used to found and continuously refresh breeding programs [23, 24]. They exhibit substantial levels of genetic variation which can affect the outcome and interpretation of biomedical studies [25–28]. Understanding of the contribution of this variation to phenotypes is lagging behind in Cynomolgus monkeys compared to the knowledge about human genetic and genomic variation [23]. Genome-wide catalogs of SNPs start to emerge for Cynomolgus monkeys with more and more genome sequencing projects published [21, 29–32]. However, information on structural variants, such as CNVs, is not available for Cynomolgus monkeys despite their prominent role in phenotypic variation. In this study, we comprehensively assess for the first time genome-wide copy number variation among Cynomolgus monkeys from cohorts used in pharmaceutical studies using a custom 4.2 million probes comparative genomic hybridization (CGH) array. To investigate the potential functional implications of the detected copy number variation, we used a Cynomolgus monkey specific gene expression microarray to associate CNV genotypes with expression changes of proximal genes using a *cis* expression quantitative trait loci (*cis-*eQTL) mapping approach.

## Results

### CNVs in diverse Cynomolgus monkey populations

**Fig. 1:**
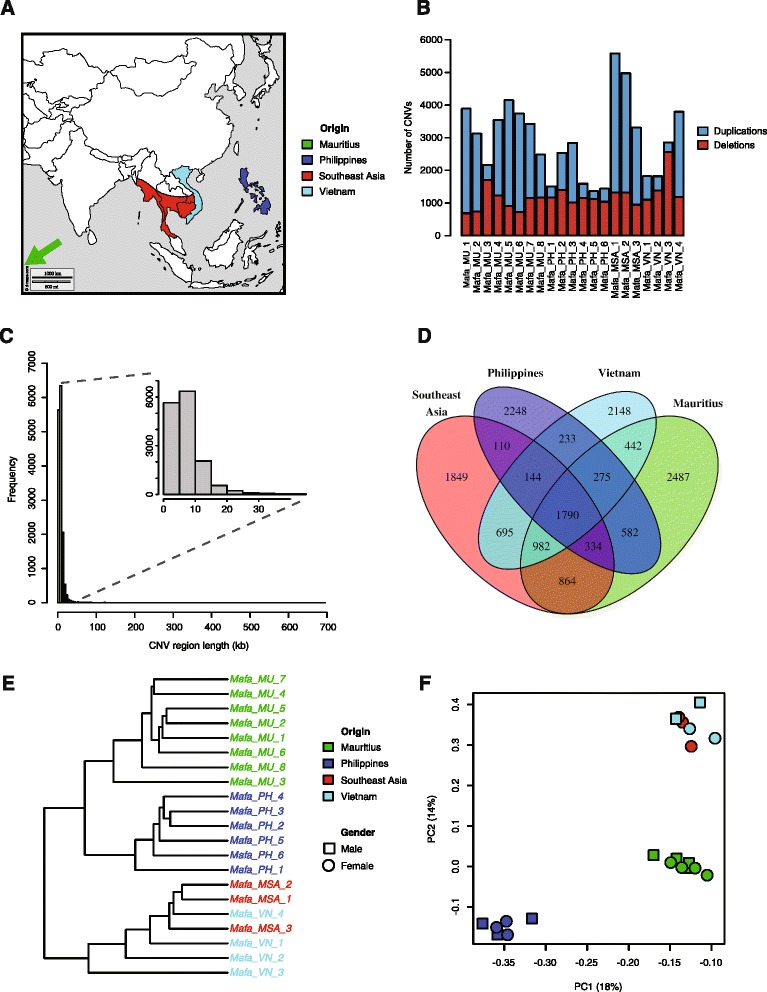
CNV genotypes. **a** Geographic origin of four natural populations, from where the tested Cynomolgus monkeys were caught. **b** Number of duplications and deletions detected per individual by combining three CNV calling approaches. **c** Size distribution of the inferred CNV regions across all 21 individuals (*n* = 15,183). **d** Number of CNV regions detected among and across the four populations. **e** Hierarchical clustering of the log_2_ − ratio genotypes of all CNV regions (*n* = 15,183) in 21 individuals. **f** Loadings of the first and second principal component based on a PCA performed on the log_2_- ratio genotypes of all CNV regions with deletions (*n* = 8495) in 21 individuals

Following the recent sequencing of the Cynomolgus monkey genome [21], we aimed at investigating CNVs among Cynomolgus monkey cohorts used in pharmaceutical research. We performed comparative genomic hybridization (CGH) using a Cynomolgus monkey specific high-resolution oligonucleotide tiling array with 4.2 million probes spanning the genome with a median spacing of 598 bp. Germline DNAs from 21 animals with different origin (Fig. 1a) were tested against a Cynomolgus monkey reference. The array data were normalized using the NimbleGen DEVA software supplemented with corrections for the GC-content [7] of the probes and for wave artifacts along the chromosomes [33]. We used three different CNV calling methods (R-GADA, DNAcopy, CopyMap) to detect high confidence CNVs [34–36]. The resulting CNV profiles were then merged per individual and only CNVs called by at least two methods were included in our analysis. We detected between 1,364 and 5,583 (mean = 2,951, SD = 1,150) autosomal CNVs per individual with on average slightly more duplications (1,758) than deletions (1,192) (Fig. 1b, Additional file 1: Table S1). To enable genotyping across individuals, we merged the overlapping CNV calls between animals into CNV regions (CNVRs). CNVs on the sex chromosomes were not considered in this analysis due to the lack of the Y-chromosome in the reference genome and because of the bias resulting from comparative genomic hybridization of female specimens relative to a male reference, lower probe density, and greater mapping uncertainty for these regions in the current assembly. We inferred a total of 15,183 CNVRs, ranging from 2.3 to 693 kb in length with a median length of 8.4 kb (SD = 15.1 kb) (Fig. 1c). In total, these CNVRs cover ~4% (~127 Mb) of the autosomal Cynomolgus monkey genome (Additional file 1: Table S2). Interestingly, 58% of CNVRs were detected in only one population, however 76% (44% of total) of them were private to one individual. 19% of CNVRs were found in two, 11% in three and 12% in all four populations (Fig. 1d).

To minimize genotyping errors we then calculated “quantitative genotypes”, defined as an individual’s median log_2_-ratio of all CGH probes within a given CNVR, and used these for all further analyses (Additional file 2: Table S3). To shed light on the relationship between individual monkeys and their CNV profiles, we used unsupervised hierarchical clustering and principal component analysis (PCA). Hierarchical clustering of the CNV signals showed a clear grouping of the samples by geographical origin (Fig. 1e). PCA revealed also a clear separation of the individuals by their geographical origin, particularly in monkeys originating from Mauritius (Additional file 1: Figure S1). Interestingly, this finding was mainly driven by the second principal component, whilst the first principal component correlated with the duplication/deletion ratio of individual monkeys (Fig. 1b). We assumed different population genetic properties of duplications and deletions, similar to previous observations in great apes and humans [37, 38]. Therefore we performed PCA on deletions and duplications separately to assess which structural variant serves as more reliable population genetic marker. In this analysis deletion genotypes segregate most accurately Cynomolgus monkeys according to the different island and mainland populations (Fig. 1f and Additional file 1: Figure S1).

### Gene expression and CNVs

**Fig. 2:**
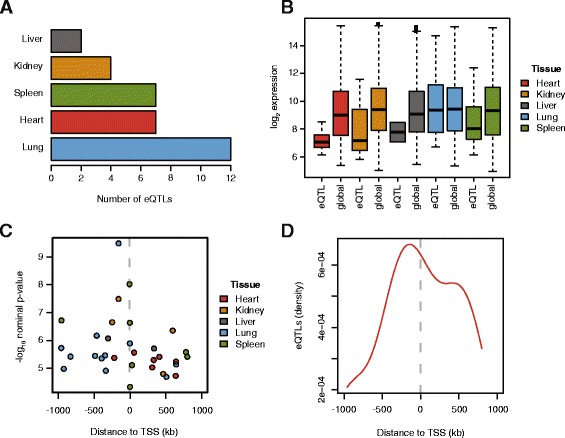
eQTLs. **a** Number of detected *cis*-eQTL per tissue under 10% false discovery rate (FDR). **b** Average expression levels of eQTL genes in each tissue versus the average expression level of all genes in the respective tissue. **c** Nominal *p*-value of all detected *cis*-eQTL as a function of the distance to the transcription start site (TSS) of the eQTL CNV to its associated gene. **d** Density of detected *cis*-eQTLs as a function of the distance to the transcription start site (TSS) of the eQTL CNV to its associated gene

Next we assessed the effect of CNVs on gene expression. Using Cynomolgus monkey specific transcriptome-wide gene expression arrays, we measured the expression levels of 18,280 genes in five vitally important tissues (liver, spleen, lung, heart, kidney) from the same 21 animals. We associated the expression levels of each gene with CNVRs residing within 1 Mb of the transcription start site (TSS) for each tissue separately. Only CNVs detected in at least two individuals (*n* = 7,266) were used for this *cis* expression quantitative trait loci (*cis-*eQTL) analysis. Genome-wide eQTL mapping was performed using fastQTL [39] and correction for multiple testing was carried out in two steps. First local permutations were applied to correct for multiple variants per gene [39] and then false discovery rate (FDR) was calculated per tissue to account for multiple tested genes. Applying an FDR cutoff of 10%, we mapped a total of 32 *cis-*eQTL genes across all five tissues, ranging from two to twelve *cis-*eQTLs per tissue (Fig. 2a, Additional file 1: Table S4). Closer inspection of eQTL genes revealed in general lower average expression levels than the tissue average (Fig. 2b), however this difference was only significant in heart (Wilcoxon rank sum test, *p* = 0.005). Functional annotation using the DAVID knowledgebase [40] showed that half of the eQTL genes encode for membrane proteins and that most eQTL genes are involved in processes like metabolism, receptor activity, and transcription. Moreover ~2/3 of the eQTL genes are associated with metabolic or cardiovascular diseases (Additional file 1: Table S4).

Closer analysis of the CNV-eQTL associations revealed increasing significance and frequency for CNVs in close proximity to the transcription start site (TSS) (Fig. 2c). The highest density of eQTL CNVs was found around 200 kb upstream of the TSS (Fig. 2d), which usually marks DNaseI hypersensitive regulatory DNA regions [41]. However, eQTL CNVs were generally not found to be located more often upstream of the TSS than downstream (Binomial test, *p* = 0.56).

Previous research suggests that CNVs might affect gene expression changes of multiple proximal genes [4]. We therefore sought to discover additional, weaker associations within the detected eQTL regions, which we might have missed in the genome-wide eQTL mapping. For each eQTL region, all associations between genes within 1 Mb of an eQTL gene’s TSS and the eQTL CNV were assessed and corrected for multiple testing per region by Bonferroni correction. A total of 12 additional associations were detected in ten out of 31 non-overlapping eQTL regions. Within these regions, on average 11.3% of genes were also associated with the eQTL CNV and all associations showed the same directionality as the eQTL.

**Fig. 3:**
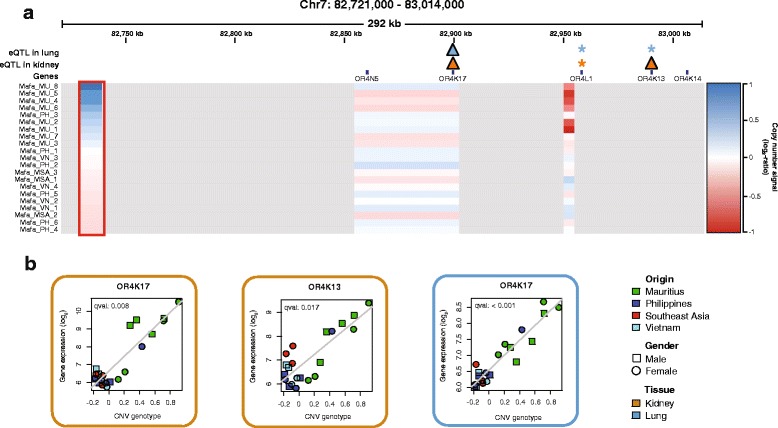
eQTL loci. **a** region on chromosome 7 containing olfactory receptor *cis*-eQTLs in both kidney (orange) and lung (blue). CNV loci are color coded according to the copy number signal (copy number gain in blue, copy number loss in red). The red box highlights the CNV locus, which shows duplication events associated with gene expression changes of proximal olfactory receptor genes. Triangles indicate an association reported from the genome-wide *cis*-eQTL mapping, while stars indicate additional associations revealed by the eQTL region analysis. **b** eQTL association plots for olfactory receptor *cis*-eQTLs on chromosome 7 in both kidney (orange) and lung (blue). CNV genotype refers to the quantitative genotype defined as the median log_2_-ratio of CGH probes contained within the CNV

**Fig. 4:**
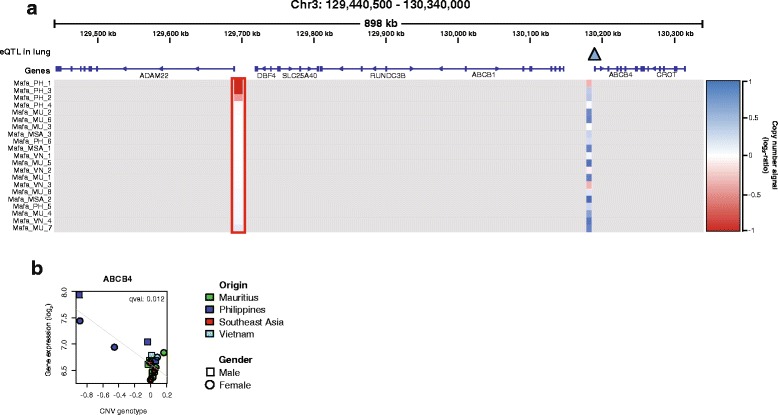
eQTL loci. **a** Region on chromosome 3 containing the *ABCB4 cis*-eQTL in lung (blue triangle). CNV loci are color coded according to the copy number signal (copy number gain in blue, copy number loss in red). The red box highlights the CNV locus, which shows deletion events associated with gene expression changes of the *ABCB4* gene. **b** eQTL association plots of *ABCB4* expression levels with the deletion CNV found in individuals from the Philippines. CNV genotype refers to the quantitative genotype defined as the median log_2_-ratio of CGH probes contained within the CNV

Among the most significant associations we found a group of olfactory receptor (OR) genes (*OR4K17, OR5M9*) on Cynomolgus monkey chromosome 7 and 14 as well as the ATP-binding cassette transporter 4 (*ABCB4*) on chromosome 3, also known as multidrug resistance protein 3 (*MDR3*) (Additional file 1: Table S4). In close proximity to the OR genes on chromosome 7 we detected a duplication event associated with expression changes of *OR4K17* in kidney and in lung and *OR4K13* in kidney (Fig. 3, Additional file 1: Figure S2). Further investigation of this eQTL region revealed additional associations with *OR4K13* in lung and *OR4L1* in both kidney and lung. For *ABCB4* we detected a deletion ~480 kb upstream of its TSS associated with increased transporter expression in lung (Fig. 4, Additional file 1: Figure S3). Both examples highlight the impact of intergenic CNVs on gene expression in a tissue specific manner.

## Discussion

Taking advantage of the recently sequenced Cynomolgus monkey genome, we developed species-specific microarrays for CNV detection and transcriptome profiling to explore CNV diversity, genome variation, and transcriptional consequences in natural monkey populations. For this approach we developed a computational pipeline combining careful data normalization and 3 different, non-redundant CNV calling methods enabling us to assess copy number variation in Cynomolgus monkeys at very high resolution (>2 kb). According to our knowledge, this is the first study to investigate natural copy number variation in this species.

We predominately find small CNVs among our individuals with a median length of about 8 kb. Technically, this finding highlights the importance of a meticulous CNV calling approach, since we operate close to the resolution limit of the array with many CNVs encompassing only 5 probes. Biologically, this finding is in line with current human genetic research, which suggests that individuals from normal and healthy populations carry mostly short CNVs (median length of 7.4 kb) [38]. Genome-wide mapping of CNVs to copy number variable regions revealed that more than half of all CNVs were population specific with a majority detected in only one individual, suggesting that many of the population specific CNVs are probably found at low frequency at the respective loci. Genotyping of Cynomolgus monkeys based on CNVs revealed a clustering by origin, clearly separating the Mauritian and Philippine island populations from mainland populations, an effect probably mediated by genetic bottlenecks and geographical isolation. For example Mauritian monkeys are descendants from a small number of individuals brought to the island in the 16th century [31]. Separation between the Southeast Asian and Vietnamese mainland populations is less pronounced, in line with the fact that these two populations share geographically adjacent biotops [23]. In this context we also discovered that duplications are less informative than deletions as population genetic markers in agreement with CNV profiling studies in great apes and humans [37, 38]. These studies have shown that deletions are less likely than duplications to be subjected to recurrent mutation events. Hence deletions cannot change their copy number state so dynamically over a short time period and are more likely to exhibit identity by descent as a result of an ancestral mutation event.

Since the median gene locus size in Cynomolgus monkeys is 47 kb, the majority of CNVs are too small to delete or alter the copy number of an entire gene making them less likely to become an evolutionary constraint. Furthermore, short CNVs are more frequently generated de novo than large CNVs (>500 kb) [42], which indicates that they are not under strong purifying selection in contrast to deleterious large CNVs [19]. Therefore the genomic regions where most CNVs are found might be those where copy number is less important or where CNVs affect regulatory regions (enhancers, insulators, silencers) that have modulatory impact on gene expression. We find that a relatively small number of CNV loci are associated with gene expression changes, suggesting that most copy number variation have no link to gene expression regulation, similar to single nucleotide variants. When looking at the association strength of our eQTLs, it became evident that the strongest associations are detected with variants close to their targets’ TSS. This relationship between genetic distance to TSS and stronger association effects appears to be similar between CNVs and SNPs. However eQTL-SNPs distribute more symmetrically in very close proximity to the TSS (within +/− 100 kb) [13, 43, 44], whilst our eQTL-CNV distributions peak ~200 kb upstream of the TSS. Therefore it is possible that eQTL-CNVs indicate more tissue-specific regulatory DNA regions or enhancer elements, whilst the majority of eQTL-SNPs mark general, tissue-independent regulatory elements which tend to be found in very close proximity to the TSS [45, 46]. Indeed, almost all of the CNV-eQTLs identified in our study exhibit tissue-specific effects. Furthermore, the eQTL-genes show generally lower expression levels than the respective tissue average. This observation might indicate, that highly expressed genes are either depleted of regulatory variants by purifying selection or that gene regulatory networks can buffer the effects of regulatory variants. However, lower expressed genes with insufficient network mediated buffering might be more responsive to CNVs in regulatory DNA regions. The resulting change in expression level might offer an adaptive advantage in a tissue-specific manner. In this respect, we highlight two particularly interesting eQTL regions on Cynomolgus monkey chromosome 7 and 3. A duplication event on chromosome 7 changes the expression levels of a group of OR genes in lung and kidney from a very low level to a level close to the average global gene expression. Although olfactory receptors are typically not expected to be expressed in internal organs, they belong to genes relevant to the immediate environment and have been found overrepresented amongst copy number variable genes [47]. Recent studies highlighted a role of such receptors in kidney where their chemosensory function plays a role in tracking of the chemical composition of blood and tubular fluids and the modulation of renin secretion, blood pressure, and glomerular filtration rate [48, 49]. On chromosome 3 we detected a deletion event associated with increased expression of *ABCB4* in lung. This gene product is known to act as tumor suppressor once overexpressed in lung cancer [50]. Since *ABCB4* expression was shown to be regulated by epigenetic silencing, a deletion 480 kb upstream of this gene might abolish epigenetic control of transcription, possibly resulting in constitutive overexpression. Carrier of such copy number variations modulating expression levels of a tumor suppressor gene might benefit from protective advantages.

## Conclusion

We describe for the first time substantial copy number variation in natural *Macaca fascicularis* populations as an additional genetic source of diversity and interindividual variation. We report several tissue specific associations between CNVs and expression levels of proximal genes providing a molecular link for variable transcriptional programs between individuals. As an example a genomic region on chromosome 7, which harbors several OR genes, shows an association between gene expression and a close-by duplication event in both kidney and lung. Our data suggest that CNVs shape tissue transcriptomes of vitally important organs, possibly offering an adaptive advantage.

## Methods

### Ethics statement

All peripheral blood and tissue samples (heart, kidney, liver, lung, spleen) used in this study were supplied by AAALAC accredited contract research organizations. Animal samples were taken from healthy, untreated animals of GLP drug-safety studies in accordance with local, national and international regulations. All procedures were approved by the Institutional Animal Care and Use Committee (IACUC) and governmental agencies responsible for animal welfare in compliance with local laws and regulations.

### Animal samples

Tissue samples for CGH and expression analysis were taken from Cynomolus monkeys obtained from breeding centers located in the Philippines (3 females and 3 males), in Vietnam (2 males, 2 females), in China for animals from Mainland Southeast Asia (3 females), or in Mauritius (4 females and 4 males) (Fig. 1a). Blood samples for CGH analysis were taken from Cynomolgus monkeys with Mauritian origin (25 males). Details (gender, weight, age, origin) of all animals and their suppliers are on record and were part of the data submitted to public databases.

### NimbleGen gene expression analysis

Cynomolgus monkey tissues were homogenized in tubes prefilled with 1.4 mm ceramic beads and QiaGen’s lysis reagent RLT using a FastPrep-24 instrument (MP Biomedicals, Solon, OH, USA). Total RNA from lysates was extracted using the RNeasy Mini kit combined with DNase treatment on a solid support (Qiagen Inc., Valencia, CA, USA). RNA quality assessment and quantification was performed using microfluidic chip analysis on an Agilent 2100 bioanalyzer (Agilent Technologies Inc., Santa Clara, CA, USA). On a Biomek FXp workstation (Beckman Coulter Inc., Brea, CA, USA), 10 ng of total RNA was used to prepare cDNA with the NuGen Ovation Pico WTA System V2 (NuGEN Technologies, Inc., SanCarlos, CA, USA), followed Cy3 labeling of cDNA with the Roche NimbleGen One Color DNA Labeling Kit. NimbleGen 12x135K gene expression microarrays (design: 120419_Cynomolgus_v5_TH_exp_HX12) were hybridized with 4 μg of Cy3-labeled cDNA for 16 h at 42 °C and were washed and dried according to the manufacturer’s instruction. Microarray data were collected by confocal scanning using the Roche NimbleGen MS200 Microarray scanner at 2 μm pixel resolution (Roche NimbleGen, Inc., Madison, WI, USA). NimbleGen probe intensities were subjected to Robust Multi-Array Analysis (RMA) with background correction and quantile normalization as implemented in the NimbleScan Software, version 2.6 (Roche NimbleGen, Inc., Madison, WI). Averaged gene-level signal intensities were summarized into gene calls and log_2_ transformed. To summarize the major biology of genes, functional annotation was performed using the DAVID knowledgebase version 6.8 (https://david.ncifcrf.gov/).

### Comparative genomic hybridization arrays

Cynomolgus monkey spleen tissues were homogenized in tubes prefilled with 1.4 mm ceramic beads and QiaGen’s lysis reagent ALT using a FastPrep-24 instrument (MP Biomedicals, Solon, OH, USA) and then incubated with Proteinase K at 55 °C for 1 h followed by RNAse A treatment at 25 °C for 2 min (Qiagen Inc., Valencia, CA). Cynomolgus monkey blood specimens (200 μl) were incubated at 70 °C for 10 min in QiaGen’s lysis reagent ALT with Proteinase K and RNAse A. Genomic DNA from lysates was extracted using the QIAamp Mini kit (Qiagen Inc., Valencia, CA, USA). Assessment of unfragmented, high molecular weight DNA and quantification was performed using microfluidic chip analysis on an Agilent 2100 bioanalyzer (Agilent Technologies Inc., Santa Clara, CA, USA). 0.5 μg of DNA from one animal tissue at a time and 0.5 μg of reference DNA - pooled DNA from blood specimens of 25 male Cynomolgus monkeys — were used for labeling by an isothermal Klenow fill-in reaction with either Cy3 or Cy5 random nonamer primer using the Roche NimbleGen Dual color labeling kit (Roche NimbleGen, Inc., Madison, WI). Labeling Hybridization Controls were spiked-in as quality controls for copy number variation detection (Roche NimbleGen, Inc., Madison, WI).

NimbleGen 4.2 M CGH microarrays (design: 120405_Cynomolgus5_CGH_UX1) were hybridized with 34 μg of Cy3- and 34 μg of Cy5-labeled DNA for 72 h at 42 °C. After hybridization, microarrays were washed and dried according to the manufacturer’s instruction, whereat 150 mM 1,3,5-Triaza-7 phospha-adamantane was included in the last washing step to avoid interference of ozone with the Cy5 dye during drying and scanning. Microarray data were collected by confocal scanning using the Roche NimbleGen MS200 Microarray Scanner at 2 μm pixel resolution (Roche NimbleGen, Inc., Madison, WI, USA).

### aCGH normalization

aCGH probe intensities were subjected to LOESS spatial correction, background correction, and q-spline normalization as implemented in the NimbleGen DEVA software, version 1.2 (Roche NimbleGen, Inc., Madison, WI). The data were then additionally normalized for probe GC-content [7]. To estimate the effect of probe GC-content on the measured log_2_-ratios, linear models were fitted for each array according to following formula:

$$ { \log}_2\left({R}_i\right)=\alpha +{\beta}_1 G{C}_i+{\beta}_2 G{C}_i^2+{\varepsilon}_i $$

Where log_2_(*R*_*i*_) is the measured log_2_ − ratio of an aCGH probe *i*, *a* the intercept, *GC*_*i*_ the probes GC content and *ε*_*i*_ a random error. The estimated effect of the probe GC-content was then subtracted from the measured log_2_ − ratios (i.e. residualized). Furthermore the data were normalized for wave artifacts along chromosomes as described by [33]. This was done by fitting a local regression (LOESS) model for each chromosome and array separately to estimate the effect of chromosomal position on the measured log_2_ − ratios:

$$ { \log}_2\left({R}_i\right)= g\left( po{s}_i\right)+{\varepsilon}_i $$

Where *g* is the local regression function, *pos*_*i*_ denotes the position of the probe *i* on the chromosome and *ε*_*i*_ a random error. Because the fraction of the data used in each local window (neighborhood) during model fitting is a crucial parameter, the normalization was performed across different fractions. The best was then selected based on signal-to-noise ratio (SNR) improvements before and after normalization using a CNV test set. The test set consisted of CNVs called based on the probe GC-content normalized data using all three callers with standard settings and the results were processed in the same way as the final CNV calls (see: CNV calling). Only CNVs detected in at least 2 individuals were retained for more confident CNV calls. For each individual, the signal-to-noise ratio of each aCGH probe in each CNV of the test set was calculated in the following way:

$$ S N{R}_i=\frac{\left|{ \log}_2\left({R}_i\right)\right|}{\sigma_{Ci}} $$

*SNR*_*i*_ denotes the signal-to-noise ratio of a given probe *i*, and *σ*_*Ci*_ the standard deviation of all probes on the same chromosome as probe *i*. The average SNR of all CNV probes per CGH array was used as metric to evaluate the normalization performance. Using this approach, a fraction of 4000 probes per model fitting step resulted in the largest median SNR improvement (1.1%) and was therefore chosen (Additional file 1: Figure S4, Table S5).

### CNV calling

CNV calling was performed with 3 inherently different approaches to mitigate method specific errors: R-GADA [34] was used with the following parameters: alpha = 0.2, T = 4.5, minseglen = 5. DNAcopy [35] was used with minseglen = 5, undosd = 3, undoprune = 0.05 and data smoothing was applied prior to CNV calling. CopyMap [36] was used with r = 20, T = 4, m = 5, a = 2.1, *P* = 0.001. Furthermore, for R-GADA and DNAcopy z-scores were calculated for all CNV calls based on the mean log_2_ − ratio of the CNV, and only CNVs with z-scores >1.5 or <−1.5 retained. For CNVs called by CopyMap a carrier probability of at least 0.8 was required.

The three obtained CNV calling profiles per individual were then merged and only CNVs called by at least two methods were kept, and loci with conflicting copy number states were removed. These resulting profiles were then further merged between individuals to obtain CNV regions that could be genotyped across individuals. In cases where an individual carried more than one CNV in a CNV region, the locus was marked as a complex locus and removed from subsequent steps. Additionally CNV loci located on the X chromosome or within array probe gaps larger than 500 kb + − 250 kb (e.g. centromeres) were removed. To avoid potential calling mistakes the median log_2_ − ratio of each CNV was used as genotype rather than the discrete copy number state provided by the CNV calling methods. PCA was performed with the R-function princomp to visualize and evaluate the inferred CNV genotypes together with hierarchical clustering of the Pearson correlation between log_2_ − ratios of samples.

### eQTL analysis

To assess the potential functional impact of copy number variants, we associated the inferred CNV genotypes with the expression level of proximal genes in each of the five tissues by using a *cis* expression quantitative trait loci (*cis-*eQTL) approach. No complex CNV loci were used for that purpose and in order to avoid outlier driven results only CNVs called in at least two individuals were retained. The expression level of each gene was tested for associations with all CNVs within 1 Mb of its transcription start site (TSS) using following linear model:

$$ {E}_{i t}=\alpha +{\beta}_1{C}_i+{\beta}_2{G}_{i t}+{\varepsilon}_{i t} $$

Where *E*_*it*_ is the measured expression level of a gene *E* in tissue *t* for the *i* ^th^ individual, *C*_*i*_ is the genotype of a proximal CNV *C* for the *i* ^th^ individual and *ε*_*it*_ a random error. To account for systematic confounding variation between samples, the loadings of the first principal component for individual *i* for the expression levels of all genes of the same tissue (*G*_*it*_) was added as covariate. This was done to account for global changes of the transcriptome resulting from batch effects, population stratification or metabolic state of an individual, which could lead to spurious associations. Multiple combinations of principal component covariates were tested and inspection of the results (data not shown) revealed the most credible eQTLs when using the loadings for the first principal component for gene expression levels as covariate. An adapted version of the fastQTL software [39] was used to test all possible associations using this model. Correction for multiple testing was carried out in two steps, where first local permutations were applied to correct for multiple variants per gene [39] and then the false discovery rate (FDR) (q-value R-package, Storey J., 2015) was calculated per tissue to account for multiple tested genes. Only eQTLs below an FDR of 10% were considered as significant.

To further investigate the impact of CNVs on the gene expression landscape, genes within the regions of detected eQTLs were investigated for further associations with the eQTL CNV. The expression levels of all genes within 1 Mb from the TSS of an eQTL gene were tested for an association with the eQTL CNV with the same linear model as used for eQTL mapping. Bonferroni correction was calculated for all tested associations per region and association with a corrected *p*-value <0.05 were considered significant.

## Additional files

Additional file 1: Figures S1 – S4 and Tables S1, S2, S4, S5. Supplemental figures and tables on CNV calling and eQTL mapping. (DOCX 1151 kb)
Additional file 2: Table S3. Quantitative genotypes for all 15,183 inferred CNV regions (CNVRs). chr represents the chromosome on which the CNVR is located, while start and end indicate the coordinates of the first respectively last CGH probe contained in the CNVR. length_kb is the estimated CNVR length in kilobases. CNV_detection_status indicates whether a CNVR contains only deletions (del), duplications (dup) or both (cnv) and eqtl_mapping indicates whether a given CNVR was detected in at least two individuals and was therefore used for *cis*-eQTL mapping (1) or not (0). The columns thereafter are the quantitative genotypes defined as an individual’s median log_2_-ratio of all CGH probes within a given CNVR. (XLSX 5415 kb)
