# Supplementary material for: Diversity and regulatory impact of copy number variation in the primate Macaca fascicularis
Source: BMC Genomics. 2017 Feb 10;18:144. doi: 10.1186/s12864-017-3531-y (PMC5301398; doi:10.1186/s12864-017-3531-y)
Supplement: Additional file 1: Figures S1 – S4 and Tables S1, S2, S4, S5. — Supplemental figures and tables on CNV calling and eQTL mapping. (DOCX 1151 kb) [file 12864_2017_3531_MOESM1_ESM.docx]

**Table S1: Number of detected CNVs:**  Number of CNVs detected per individual by the three CNV calling methods prior to merging of individual CNV profiles calls into CNV regions across individuals. N=21 samples.

| **Sample** | **Duplications** | **Deletions** | **Total CNVs** |
| --- | --- | --- | --- |
| Mafa_MSA_1 | 4262 | 1321 | 5583 |
| Mafa_MSA_2 | 3649 | 1327 | 4976 |
| Mafa_MSA_3 | 2361 | 948 | 3309 |
| Mafa_MU_1 | 3205 | 687 | 3892 |
| Mafa_MU_2 | 2385 | 741 | 3126 |
| Mafa_MU_3 | 463 | 1700 | 2163 |
| Mafa_MU_4 | 2316 | 1232 | 3548 |
| Mafa_MU_5 | 3242 | 911 | 4153 |
| Mafa_MU_6 | 3020 | 719 | 3739 |
| Mafa_MU_7 | 2264 | 1158 | 3422 |
| Mafa_MU_8 | 1311 | 1170 | 2481 |
| Mafa_PH_1 | 340 | 1170 | 1510 |
| Mafa_PH_2 | 1128 | 1402 | 2530 |
| Mafa_PH_3 | 1826 | 1015 | 2841 |
| Mafa_PH_4 | 438 | 1151 | 1589 |
| Mafa_PH_5 | 237 | 1127 | 1364 |
| Mafa_PH_6 | 404 | 1038 | 1442 |
| Mafa_VN_1 | 725 | 1099 | 1824 |
| Mafa_VN_2 | 440 | 1378 | 1818 |
| Mafa_VN_3 | 291 | 2563 | 2854 |
| Mafa_VN_4 | 2612 | 1185 | 3797 |

**Table S2: CNVRs per chromosome** Number of CNVRs per chromosome, average and total CNVR length, and the percentage of the chromosome covered by CNVRs**.**

| **chromosome** | **#cnvs** | **avg length**  **(kb)** | **total length**  **(kb)** | **chromosome length (kb)** | **cnv of chromosome (%)** |
| --- | --- | --- | --- | --- | --- |
| chr1 | 1364 | 8.2 | 11180.10 | 229594.24 | 4.87 |
| chr2 | 934 | 7.22 | 6747.72 | 190805.71 | 3.54 |
| chr3 | 1048 | 9.74 | 10209.26 | 197221.78 | 5.18 |
| chr4 | 871 | 7.71 | 6719.41 | 168762.93 | 3.98 |
| chr5 | 918 | 7.37 | 6769.15 | 183220.12 | 3.69 |
| chr6 | 888 | 7.95 | 7059.39 | 179267.54 | 3.94 |
| chr7 | 924 | 9.04 | 8355.83 | 170755.60 | 4.89 |
| chr8 | 794 | 8.21 | 6521.40 | 148375.95 | 4.40 |
| chr9 | 756 | 8.74 | 6606.99 | 133871.80 | 4.94 |
| chr10 | 677 | 8.02 | 5427.16 | 95118.95 | 5.71 |
| chr11 | 725 | 8.23 | 5969.60 | 135088.50 | 4.42 |
| chr12 | 498 | 7.68 | 3823.91 | 106987.73 | 3.57 |
| chr13 | 827 | 8.85 | 7317.67 | 138727.24 | 5.27 |
| chr14 | 734 | 8.43 | 6188.49 | 134024.31 | 4.62 |
| chr15 | 642 | 8.77 | 5631.26 | 110686.22 | 5.09 |
| chr16 | 607 | 9.48 | 5755.54 | 78971.47 | 7.29 |
| chr17 | 508 | 7.80 | 3963.91 | 94759.12 | 4.18 |
| chr18 | 380 | 7.79 | 2958.90 | 73889.72 | 4.00 |
| chr19 | 556 | 9.80 | 5448.26 | 65320.79 | 8.34 |
| chr20 | 532 | 7.76 | 4126.15 | 88198.65 | 4.68 |
| total | 15183 | 8.35 | 126780.1 | 2879306.38 | 4.40 |

**Table S4: genome-wide eQTL mapping results** All detected cis-eQTL associations across all tissues are listed. Functional gene annotations and genetic associations with disease (GAD) classes (https://geneticassociationdb.nih.gov/) are indicated. Nominal p-value denotes the uncorrected p-value obtained by the linear model, while beta p-value is the beta approximated permutation value obtained by fastQTL. Q-value is the FDR corrected beta p-value.

| **tissue** | **gene id** | **gene symbol** | **functional gene annotation** | **variants tested** | **cnv id** | **dist to TSS** | **nominal p-value** | **slope** | **beta p-value** | **q-value** |
| --- | --- | --- | --- | --- | --- | --- | --- | --- | --- | --- |
| heart | HP_001126102 | HP | metabolism, metabolic GAD, cardiovascular GAD | 2 | chr20_71248715 | 637802 | 1.86E-05 | -5.23904 | 1.10E-05 | 0.045313163 |
| heart | TMPRSS11E_014058 | TMPRSS11E | - | 3 | chr5_61060708 | -219549 | 4.22E-06 | -1.02858 | 1.13E-05 | 0.045313163 |
| heart | HOPX_139212 | HOPX | transcription | 6 | chr5_73528649 | 330121 | 5.04E-06 | -1.10353 | 2.73E-05 | 0.068641859 |
| heart | C1QTNF3_030945 | C1QTNF3 | cardiovascular GAD | 6 | chr6_34920268 | 643101 | 5.74E-06 | -2.49372 | 3.96E-05 | 0.085436534 |
| heart | ODF1_024410 | ODF1 | metabolism, metabolic GAD | 6 | chr8_105576105 | 56991 | 2.71E-06 | -3.69366 | 9.99E-06 | 0.045313163 |
| heart | UGT1A6_205862 | UGT1A6 | metabolism, metabolic GAD | 5 | chr12_98452925 | 409599 | 3.87E-06 | -0.717681 | 1.20E-05 | 0.045313163 |
| heart | EHF_001206615 | EHF | transcription, metabolism, metabolic GAD | 5 | chr14_38008601 | 309249 | 9.30E-06 | -2.21862 | 2.01E-05 | 0.060527996 |
| kidney | OR4K17_001004715 | OR4K17 | receptor activity | 10 | chr7_82729888 | -159990 | 3.23E-08 | 4.27523 | 4.62E-07 | 0.007534321 |
| kidney | OR4K13_001004714 | OR4K13 | receptor activity | 10 | chr7_82729888 | -249535 | 2.21E-07 | 2.95482 | 2.12E-06 | 0.017292302 |
| kidney | C1orf190_001013615 | C1orf190 | - | 7 | chr1_49881978 | 595869 | 4.41E-07 | 1.0513 | 1.27E-05 | 0.068929539 |
| kidney | GLIPR1_006851 | GLIPR1 | metabolism | 3 | chr11_73293567 | 462506 | 1.59E-05 | -0.946824 | 2.06E-05 | 0.084206874 |
| liver | RPL28_001136135 | RPL28 | metabolism | 10 | chr19_61812296 | -306140 | 8.48E-07 | 1.28237 | 7.05E-06 | 0.053888280 |
| liver | GULP1_016315 | GULP1 | metabolism, cardiovascular GAD | 2 | chr12_52614781 | 335252 | 1.94E-06 | -0.806255 | 8.25E-07 | 0.012603638 |
| lung | CRYZL1_145858 | CRYZL1 | metabolism | 13 | chr3_11978749 | -959914 | 1.86E-06 | -1.40728 | 2.01E-05 | 0.029587992 |
| lung | ABCB4_018850 | ABCB4 | metabolism, metabolic GAD, cardiovascular GAD | 2 | chr3_129690245 | -487889 | 3.55E-06 | -1.1208 | 2.73E-06 | 0.012077783 |
| lung | CNTNAP2_014141 | CNTNAP2 | metabolic GAD, cardiovascular GAD | 4 | chr3_183849127 | -337085 | 1.22E-05 | -4.79778 | 1.34E-05 | 0.023162921 |
| lung | RPS18_022551 | RPS18 | metabolism, metabolic GAD | 5 | chr4_32749036 | -464626 | 6.71E-07 | 1.36137 | 5.13E-07 | 0.003400137 |
| lung | KIF25_005355 | KIF25 | metabolic GAD | 4 | chr4_165210711 | -829498 | 3.78E-06 | -5.58818 | 1.40E-05 | 0.023162921 |
| lung | KLKB1_000892 | KLKB1 | metabolic GAD, cardiovascular GAD | 6 | chr5_179457643 | -1783 | 1.28E-06 | -2.82113 | 3.89E-06 | 0.012880492 |
| lung | F2RL2_004101 | F2RL2 | receptor activity | 8 | chr6_73678095 | 508126 | 2.02E-05 | 0.829562 | 8.79E-05 | 0.097073120 |
| lung | OR4K17_001004715 | OR4K17 | receptor activity | 10 | chr7_82729888 | -159990 | 3.25E-10 | 2.33351 | 8.04E-09 | 0.000106599 |
| lung | ABRA_139166 | ABRA | transcription, metabolic GAD, cardiovascular GAD | 7 | chr8_108782581 | -923385 | 1.04E-05 | -1.62717 | 6.17E-05 | 0.074336995 |
| lung | RABGAP1L_001243763 | RABGAP1L | cardiovascular GAD | 5 | chr1_205287055 | -348596 | 3.45E-06 | 0.254591 | 1.22E-05 | 0.023162921 |
| lung | SCARB1_001082959 | SCARB1 | receptor activity, metabolism, metabolic GAD, cardiovascular GAD | 5 | chr11_127360260 | 642655 | 7.25E-06 | 0.709079 | 2.78E-05 | 0.036880181 |
| lung | OR6B3_173351 | OR6B3 | receptor activity | 4 | chr12_104005977 | -394555 | 4.37E-06 | -2.88373 | 6.53E-06 | 0.017305874 |
| spleen | BMPER_133468 | BMPER | metabolic GAD, cardiovascular GAD | 1 | chr3_93158281 | 781027 | 2.63E-06 | -3.4384 | 2.62E-06 | 0.010918354 |
| spleen | KLKB1_000892 | KLKB1 | metabolic GAD, cardiovascular GAD | 6 | chr5_179457643 | -1783 | 2.30E-07 | -2.43043 | 7.38E-07 | 0.004791264 |
| spleen | SPTA1_003126 | SPTA1 | metabolism, cardiovascular GAD | 4 | chr1_138046126 | 27709 | 7.69E-06 | -0.812929 | 3.68E-05 | 0.079606615 |
| spleen | C11orf85_001037225 | C11orf85 | - | 18 | chr14_8575524 | -956573 | 1.91E-07 | -4.31058 | 3.36E-06 | 0.010918354 |
| spleen | OR5M9_001004743 | OR5M9 | receptor activity | 7 | chr14_16700413 | -6666 | 9.50E-09 | -2.78729 | 9.41E-09 | 0.000122303 |
| spleen | COPB1_001144062 | COPB1 | - | 6 | chr14_58777903 | 799835 | 3.83E-06 | -0.87941 | 2.60E-05 | 0.067449916 |
| spleen | VLDLR_001018056 | VLDLR | receptor activity, metabolic GAD, cardiovascular GAD | 1 | chr15_74972410 | 0 | 4.65E-05 | -1.67083 | 5.17E-05 | 0.095968239 |

**Table S5: local eQTL mapping results** For each eQTL region in Table S4, all associations between genes within 1Mb of an eQTL gene’s TSS and the eQTL CNV were assessed and corrected for the number of tests within each eQTL region by Bonferroni correction. All additional associations that were detected across all tissues are listed. Nominal p-value denotes the uncorrected p-value obtained by the linear model and corrected p-value represents Bonferroni corrected p-values.

| **tissue** | **gene id** | **gene symbol** | **cnv id** | **dist to TSS** | **nominal p-value** | **slope** | **corrected p-value** |
| --- | --- | --- | --- | --- | --- | --- | --- |
| heart | TAT_000353 | TAT | chr20_71248715 | 131195 | 0.001211458 | -2.12502997 | 0.030286444 |
| heart | SLC1A2_004171 | SLC1A2 | chr14_38008601 | 1102982 | 4.73E-05 | -3.222420707 | 0.000992474 |
| kidney | OR4K17_001004715 | OR4K17 | chr7_82729888 | -159990 | 3.23E-08 | 4.275232684 | 1.45E-06 |
| kidney | OR4L1_001004717 | OR4L1 | chr7_82729888 | -218557 | 1.89E-05 | 1.795486829 | 0.000850908 |
| kidney | PHLDA1_007350 | PHLDA1 | chr11_73293567 | -75444 | 0.000366337 | -0.748184073 | 0.00439604 |
| liver | RPL28_001136136 | RPL28 | chr19_61812296 | -306139 | 1.71E-05 | 2.97748235 | 0.0011105 |
| liver | TFPI_006287 | TFPI | chr12_52614781 | 1082267 | 0.001712429 | -0.26955489 | 0.011987005 |
| lung | COL11A2_080679 | COL11A2 | chr4_32749036 | -383365 | 0.000101907 | 3.447280257 | 0.00397437 |
| lung | CCR6_004367 | CCR6 | chr4_165210711 | 51133 | 0.001478536 | -1.729248456 | 0.016263901 |
| lung | OR4L1_001004717 | OR4L1 | chr7_82729888 | -218557 | 4.39E-05 | 0.898413877 | 0.001712694 |
| lung | OR4K13_001004714 | OR4K13 | chr7_82729888 | -249534 | 1.66E-06 | 2.988777149 | 6.46E-05 |
| lung | DUSP28_001033575 | DUSP28 | chr12_104005977 | -876777 | 0.000109729 | 1.820346901 | 0.0023043 |

**Table S6: Wave artifact normalizations SNR** Median average signal-tonoise ratio (SNR) per sample for aCGH probes within CNVs called from probe GC-content normalized aCGH data across all tested LOESS fraction values.

| **Normalization** | **Median SNR** |
| --- | --- |
| Probe GC-content only | 1.4921 |
| 5000 probes | 1.5084 |
| 4500 probes | 1.5082 |
| 4000 probes | 1.5089 |
| 3500 probes | 1.5087 |
| 3000 probes | 1.5083 |
| 2500 probes | 1.5082 |
| 2000 probes | 1.5083 |
| 1500 probes | 1.5078 |
| 1000 probes | 1.5061 |
| 500 probes | 1.5018 |
| 100 probes | 1.4577 |
|  |  |


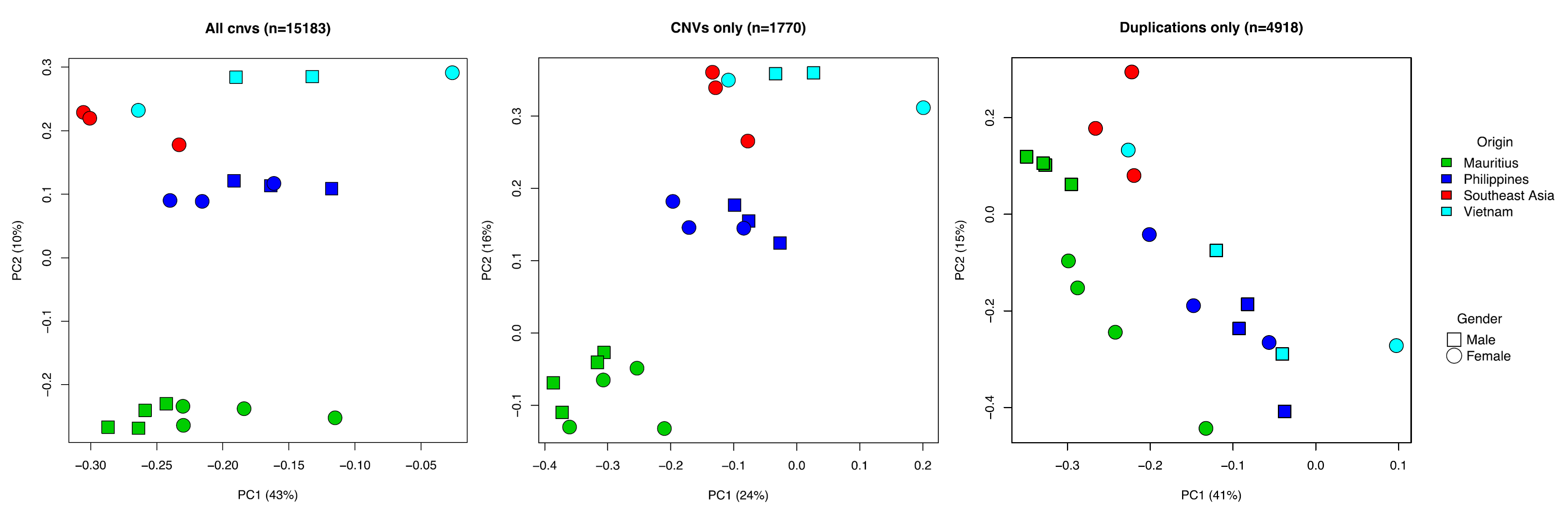


**Figure S1:** Principal Component Analysis (PCA) of CNV genotypes. Loadings of the first and second principal component (PC) based on a PCA performed on the log_2_- ratio genotypes of all CNV regions (n=15183), CNV regions with duplications and deletions (n=1770), and duplications only (n=4918) in 21 individual Cynomolgus monkeys.

**Figure S2:** Profiles of a CNV locus on chromosome 7 associated with expression changes of the OR4K17 and OR4K13 genes. CNV signals in comparison to a reference standard are displayed as log_2_-ratio along genomic positions of chromosome 7 (grey dots). Green or red bar intensity denotes higher, respectively lower median log_2_-ratio of probes within the CNV.

**Figure S3:** Profiles of a CNV locus on chromosome 3 associated with expression changes of the ABCB4 gene. CNV signals in comparison to a reference standard are displayed as log_2_-ratio along genomic positions of chromosome 3 (grey dots). Green or red bar intensity denotes higher, respectively lower median log_2_-ratio of probes within the CNV.

**Figure S4: Wave artifact normalizations SNR** Average signal-to-noise ratio (SNR) per sample for aCGH probes within CNVs called from probe GCcontent normalized aCGH data across all tested LOESS fraction values.
